# Prognostic burden of heart failure recorded in primary care, acute hospital admissions, or both: a population‐based linked electronic health record cohort study in 2.1 million people

**DOI:** 10.1002/ejhf.709

**Published:** 2016-12-23

**Authors:** Stefan Koudstaal, Mar Pujades‐Rodriguez, Spiros Denaxas, Johannes M.I.H. Gho, Anoop D. Shah, Ning Yu, Riyaz S. Patel, Chris P. Gale, Arno W. Hoes, John G. Cleland, Folkert W. Asselbergs, Harry Hemingway

**Affiliations:** ^1^ Farr Institute of Health Informatics Research London UK; ^2^ UCL Institute of Health Informatics University College London London UK; ^3^ Department of Cardiology University Medical Centre Utrecht Utrecht The Netherlands; ^4^ UCL Institute of Cardiovascular Sciences University College London London UK; ^5^ Leeds Institute of Cardiovascular and Metabolic Medicine University of Leeds Leeds UK; ^6^ Julius Centre for Health Sciences and Primary Care University Medical Centre Utrecht Utrecht The Netherlands; ^7^ Faculty of Medicine, National Heart & Lung Institute Imperial College London London UK

**Keywords:** Heart failure, Epidemiology, Prognosis, Acute hospital admission, Primary care, Electronic health records

## Abstract

**Aims:**

The prognosis of patients hospitalized for worsening heart failure (HF) is well described, but not that of patients managed solely in non‐acute settings such as primary care or secondary outpatient care. We assessed the distribution of HF across levels of healthcare, and assessed the prognostic differences for patients with HF either recorded in primary care (including secondary outpatient care) (PC), hospital admissions alone, or known in both contexts.

**Methods and results:**

This study was part of the CALIBER programme, which comprises linked data from primary care, hospital admissions, and death certificates for 2.1 million inhabitants of England. We identified 89 554 patients with newly recorded HF, of whom 23 547 (26%) were recorded in PC but never hospitalized, 30 629 (34%) in hospital admissions but not known in PC, 23 681 (27%) in both, and 11 697 (13%) in death certificates only. The highest prescription rates of ACE inhibitors, beta‐blockers, and mineralocorticoid receptor antagonists was found in patients known in both contexts. The respective 5‐year survival in the first three groups was 43.9% [95% confidence interval (CI) 43.2–44.6%], 21.7% (95% CI 21.1–22.2%), and 39.8% (95% CI 39.2–40.5%), compared with 88.1% (95% CI 87.9–88.3%) in the age‐ and sex‐matched general population.

**Conclusion:**

In the general population, one in four patients with HF will not be hospitalized for worsening HF within a median follow‐up of 1.7 years, yet they still have a poor 5‐year prognosis. Patients admitted to hospital with worsening HF but not known with HF in primary care have the worst prognosis and management. Mitigating the prognostic burden of HF requires greater consistency across primary and secondary care in the identification, profiling, and treatment of patients.

**Trial registration**: NCT02551016

## Introduction

Management of chronic diseases with acute exacerbations, such as heart failure (HF), is often fragmented across primary and secondary care.^1^, ^2^, ^3^, ^4^ Yet, most clinical trials and registries that have guided HF care and informed patients about their prognosis have had a HF‐related hospitalization as a prerequisite for study enrolment. As a result, the extent to which this evidence should be extrapolated to HF patients seen in other levels of the healthcare system, those who are never hospitalized for example, remains largely uninvestigated. Therefore, as for many diseases, external validity of HF trials and cohorts is questionable and reflects the paucity in our understanding of how evidence should be generalized into guidelines.^5^

The challenge to prognosticate heterogeneous diseases such as HF was recently reinforced by data from the Swedish healthcare system, showing that patients included in trials were poorly representative of patients with HF encountered in the general population, and approximately one in three such patients had never been hospitalized for HF.^6^ Data from Sweden and other population‐based studies from various countries are now increasingly available.^6^, ^7^, ^8^ However, to date, most studies that report on prognosis of HF produced survival estimates for HF in general,^9^, ^10^, ^11^, ^12^, ^13^, ^14^ acute hospital admissions,^15^, ^16^, ^17^, ^18^, ^19^ or stratified for cardiac systolic dysfunction^20^, ^21^, ^22^, ^23^, ^24^ (i.e. reduced‐, mid‐, or preserved range of LVEF) for example. We hypothesize that prognostication of HF can merit from strata based on level of care to allow assessment of novel patient groups, such as patients known with HF in ambulatory care (i.e. primary or outpatient secondary care) who have not been hospitalized with HF for example, or vice versa.

Electronic health records (EHRs) are now used as an integral part of routine daily practice in primary, and, to a lesser extent, in secondary care in the UK.^25^, ^26^ They provide an unprecedented amount of data available for research and, as a result, have gained increasing attention from the scientific community as well as governmental institutions.^27^ Accordingly, we linked prospectively collected data from primary care, hospital admissions, and death certificates for 2.1 million inhabitants of England in the Cardiovascular disease research using Linked Bespoke studies and Electronic Health Records (CALIBER) programme.^28^ This platform has been extensively validated for cardiovascular research.^29^, ^30^, ^31^, ^32^ Our objectives were to assess the distribution and prognostic impact of HF among patients with HF recorded in primary care (including outpatient secondary care) (PC), acute hospital admissions, or both. For these respective strata, we assessed patient characteristics, their HF management, and their prognosis in terms of 90‐day and 5‐year mortality.

## Methods

### Study design and data sources

We used a cohort study design, based on the CALIBER programme, as described previously.^28^ *Table S1* in the Supplementary material online summarizes the STROBE^33^ and RECORD^34^ checklists for reporting on observational research in routinely collected health data. Briefly, CALIBER (www.caliberresearch.org) consists of linkage of four different prospectively collected national data sources: the Clinical Practice Research Datalink (CPRD), the Myocardial Ischaemia National Audit Project (MINAP) registry, Hospital Episodes Statistics (HES), and cause‐specific mortality in the Office for National Statistics (ONS). CPRD is a research database containing anonymized electronic PC records from 11.3 million patients in 674 general practitioner (GP) practices throughout the UK (www.cprd.com).^35^ We used data from 2.13 million patients across 225 CPRD practices in England that consented to data linkage.^28^ Previous work has shown that CPRD patients are representative of the general population of the UK in terms of sex, age, ethnicity,^35^, ^36^ and overall mortality^37^, thereby validating CPRD for epidemiological research.^32^ HES is a database containing dates and diagnostic codes for all elective and emergency admissions and procedures to National Health Service hospitals in England (www.hscic.gov.uk/hes). ONS is a database containing death certificates and provides date and causes of death (www.ons.gov.uk/ons).

### Study population and definition of heart failure

We included all patients with incident HF from 1 January 1997 to 26 March 2010 (when all record sources were concurrent). The diagnosis of HF was based on Read codes for CPRD data and International Classification of Diseases (ICD)‐9 or −10 codes in HES and ONS, using a phenotyping approach previously described (details on algorithms are available on www.caliberresearch.org/portal/ and Supplementary material online, *Table S2*).^38^ We excluded patients under 30 years of age and those not registered during the study period at a CPRD practice, or whose CPRD practices did not submit data for at least 1 year before the diagnosis of HF. The study flow chart is shown in the Supplementary material online, *Figure S1*. Based on the individual distribution of HF records, we formulated four groups of interest based on the presence or absence of a HF record in each of the three data sources (Supplementary material online, *Figure S2*).

### Clinical details of heart failure

We sought additional clinical details in the PC record relevant to diagnosis and management, including LV dysfunction confirmed by cardiac imaging, an elevated BNP concentration, referral to HF care, referral to a cardiologist, the use of loop diuretics, and symptoms and signs suggestive of HF. Relevant codes to support these criteria are listed in *Table S3* in the Supplementary material online.

### Baseline characteristics

For each patient, information on demographics (i.e. age, sex, and social deprivation), cardiovascular risk factors (i.e. smoking, hypertension, diabetes, systolic blood pressure, and body mass index), blood tests (e.g. haemoglobin, creatinine, and BNP), co‐existing morbidities (COPD, cancer, and depression), and drug prescription [loop diuretics, ACE inhibitors, ARBs, beta‐blockers, and mineralocorticoid receptor antagonists (MRAs)] were all obtained from PC consultations recorded in CPRD. Risk factors and endpoints used in this study were previously defined and their phenotyping algorithms combining Read, ICD‐10, drug, and procedure codes are published online and can be found at http://www.caliberresearch.org/portal/.^30^, ^39^ Measured values such as blood pressure were based on the value closest to the first recorded diagnosis date. A patient was considered to be adherent to a medication if there was at least one prescription of the drug present within plus or minus 6 months of the HF diagnosis.

### Statistical analyses

Hazard ratios (HRs) were derived from Cox models, with time since first HF diagnosis as the time scale, adjusted for baseline age (linear), and stratified by sex and GP practice, to take clustering between practices into account. Proportionality of hazard was verified by plotting the Schoenfeld residuals. We produced Kaplan–Meier cumulative incidence curves for 90‐day and 5‐year all‐cause, cardiovascular, and HF‐specific mortality for HF patients, stratified by EHR source (Supplementary material online, *Figure S2*). In Cox models of 5‐year mortality, we used the age‐ and sex‐matched general population as the reference group. We matched patients with HF recorded in one of their EHRs in a 1:1 ratio with an age‐ and sex‐matched general population drawn from the 2.13 million study participants in CALIBER using the R package ‘MatchIt’ with caliper set at 0.15. Data were analysed using R version 3.1.2.

### Ethical approval

This study was approved by the Independent Scientific Advisory Committee of the Medicines and Healthcare products Regulatory Agency (Protocol number 14_246R). The protocol was registered at clinicaltrial.gov (NCT02551016).

## Results

### Distribution of heart failure in the general population

**Figure 1:**
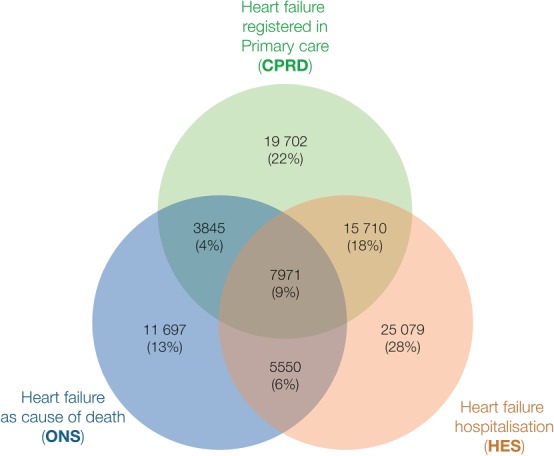
Venn diagram showing the number and percentage of records in primary care (CPRD), hospital admissions (HES), and mortality registry (ONS) for heart failure across three national sources in England, UK (n = 89 554).

Out of 2 134 615 patients in CALIBER, we identified 89 554 patients (4.2%) with a record of incident HF. The distribution of HF recorded in primary care, hospital admissions, or cause of death is shown in *Figure* 1. Out of all HF patients, 26% were recorded in primary care only, 27% in both primary care and hospital admissions, 34% in hospital admissions only, and 13% had HF as cause of death without a previous record in primary care or hospital admissions (*Figure* 1). Among patients with both HF recorded in primary care and hospital admissions, 38% were first recorded during hospitalization and the median time for an accompanying record of HF in PC was 29 days [interquartile range (IQR) 10–190 days] (Supplementary material online, *Figure S3*). Lastly, 32 338 out of 54 310 (59.5%) patients who were hospitalized for HF had a single HF‐related hospitalization episode throughout follow‐up (Supplementary material online, *Figure S4*)

### Lifestyle, cardiovascular risk factors, and co‐morbidities

Patient characteristics, stratified by EHR source, are shown in *Table* 1 and Supplementary material online *Table S4*. The median age at diagnosis for patients known in PC but not hospitalized, only hospitalized but not known in PC, or known in both levels of care was 78.8 years (IQR 70.9–85.6 years), 80.4 years (IQR 71.4–85.6 years), and 79.8 years (IQR 72.8–85.9), respectively, with roughly similar percentages of female patients present in the three respective groups (*Table* 1). Overall, cardiovascular risk factors and co‐morbidities were less common in patients with HF in PC who were never hospitalized, and the highest prevalence was observed in patients identified in HF‐related hospital admissions alone or in combination with PC, as were modifiable lifestyle factors such as smoking (*Table* 1). Missing data are listed in the Supplementary material online, *Table S5*. With regard to aetiology, HF was recorded as ‘not otherwise specified’ in CPRD in 99.8% (47 163 out of 47 228) of patients. However, the prevalence of ischaemic heart disease, which can serve as a proxy for ischaemic HF, was lowest in patients seen in PC but not hospitalized for HF [10 332 out of 23 547 (43.9%) patients], compared with 15 346 out of 30 629 (50.1%) patients acutely hospitalized without a PC record, or 13 421 out of 23 681 (56.7%) patients seen in both levels of care (*Table* 1, *P* < 0.0001). Lastly, we found that 60 042 (67.0%) patients had at least one item of additional information of which a loop diuretic prescription and signs and symptoms were most common (Supplementary material online, *Table S6*).

**Table 1:** Characteristics of patients at time of heart failure recorded in primary care, hospital admissions, or both (n = 77 857 patients)

	CPRD	CPRD and HES	HES
	Primary care record of HF but never hospitalized for HF	Primary care record of HF and hospitalized at least once for HF	Hospitalized for HF without primary care record of HF
No. of patients	23 547	23 681	30 629
Patient characteristics			
Median age (IQR) in years	78.8 (70.9–85.6)	79.8 (72.8–85.9)	80.4 (71.4–86.9)
Women (%)	12 098 (51.3%)	11 780 (49.7%)	16 005 (52.2%)
Most deprived fifth^a^	4806 (20.4%)	4312 (18.2%)	5976 (19.5%)
Current smoking^b^	2956 (14.1%)	3283 (14.9%)	5117 (19.3)
Ex‐smoker^b^	6842 (32.6%)	7339 (33.5%)	7911 (29.9%)
Never smoked^b^	11 157 (53.3%)	11 272 (51.4%)	13 425 (50.8%)
Body mass index in kg/m^2^,^b^ mean ± SD	27.1 ± 5.7	27.3 ± 5.9	27.0 ± 5.9
Systolic blood pressure in mmHg^b^, mean ± SD	142 ± 23	140 ± 24	139 ± 22
Serum creatinine, µmol/L^b^	109 ± 86	117 ± 56	113 ± 68
Haemoglobin, mmol/L^b^	13.2 ± 1.9	12.8 ± 1.9	12.8 ± 2.0
Co‐morbidity			
Hypertension^c^	17 626 (74.9%)	19 881 (84.0%)	24 600 (80.3%)
Diabetes mellitus^c^	2095 (8.9%)	3898 (16.4%)	5493 (17.9%)
Atrial fibrillation^c^	6491 (27.6%)	10 793 (45.6%)	12 432 (40.6%)
Ischaemic heart disease^c^	10 332 (43.9%)	13 421 (56.7%)	15 346 (50.1%)
Myocardial infarction^c^	4959 (21.1%)	7503 (31.7%)	8863 (28.9%)
Stroke^c^	663 (2.8%)	1047 (4.4%)	1962 (6.4%)
COPD^c^	4130 (17.5%)	5444 (23.0%)	6437 (21.0%)
Depression^c^	4854 (20.6%)	4623 (19.5%)	6383 (20.8%)
Cancer^c^	4616 (19.6%)	4569 (19.3%)	7156 (23.4%)
Heart failure medication			
Loop diuretics^d^	16 513 (70.1%)	20 911 (88.3%)	13 643 (44.5%)
ACE inhibitors/ARB^d^	13 138 (55.8%)	17 057 (72.0%)	11 844 (38.7%)
Beta‐blockers^d^	7179 (30.5%)	8190 (34.6%)	7985 (26.1%)
HF beta‐blockers^e^	3003 (12.8%)	5247 (22.2%)	3753 (12.3%)
MRAs^d^	2273 (9.7%)	5502 (23.2%)	2389 (7.8%)

CPRD, Clinical Practice Research Datalink; HES, Hospital Episodes Statistics; HF, heart failure; IQR, interquartile range; MRA, mineralocorticoid receptor antagonist; SD, standard deviation.

a Assessed by index of multiple deprivation.

b Measurement closest to and within 6 months before or on the date of HF diagnosis.

c Prior medical history of the given co‐morbidity.

d Denotes present or prescribed ± 6 months of index date for HF diagnosis.

e HF beta‐blockers include metoprolol, carvedilol, or bisoprolol.

### Heart failure management

An ACE inhibitor or an ARB was prescribed in 13 138 out of 23 547 (55.8%) PC patients who were never hospitalized but only in 11 844 out of 30 629 (38.7%) patients hospitalized for HF without a PC record, compared with the highest prescription rate in 17 057 out of 23 681 (72.0%) of patients known in both levels of care (*Table* 1). In addition, beta‐blockers were only prescribed in 7179 (30.5%), 7985 (26.1%), and 8190 (34.6%) of patients for these three groups, respectively. The relatively newest evidence‐based treatment of HF is the addition of an MRA, and these were prescribed in 2273 (9.7%) patients in the group known in PC but not acute hospitalization, in 2389 (7.8%) patients acutely hospitalized but not known in PC, compared with 5502 (23.2%) patients known in both contexts.

### Survival

**Figure 2:**
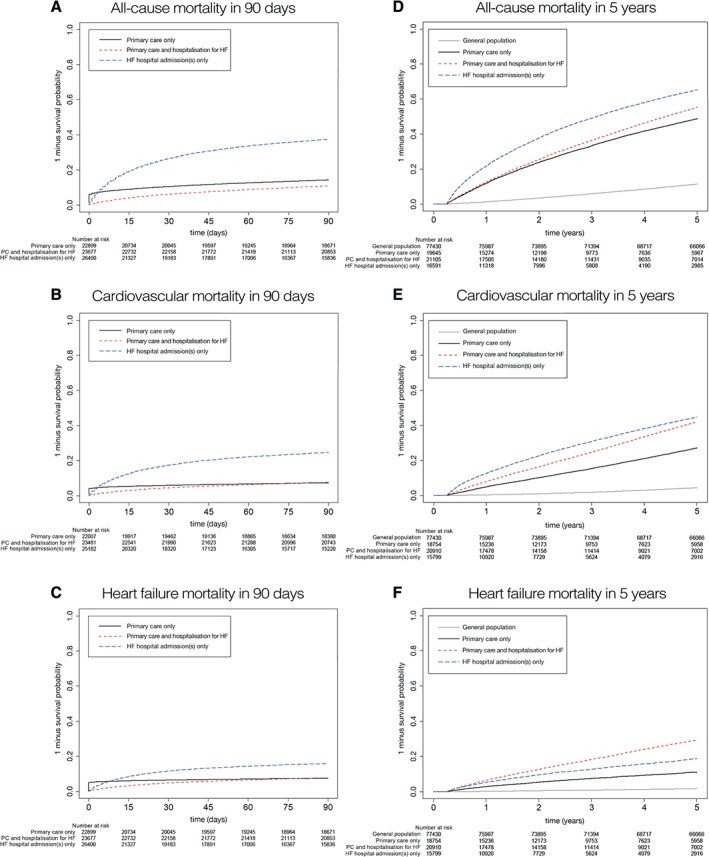
Kaplan–Meier survival curves showing the survival following heart failure (HF) recorded in primary care, acute hospital admissions, or both, for all‐cause mortality (A and D), cardiovascular mortality (B and E), and HF as cause of death (C and F). (A–C) Ninety‐day mortality; (D–F) 5‐year mortality in patients surviving the first 3 months. PC, primary care.

**Figure 3:**
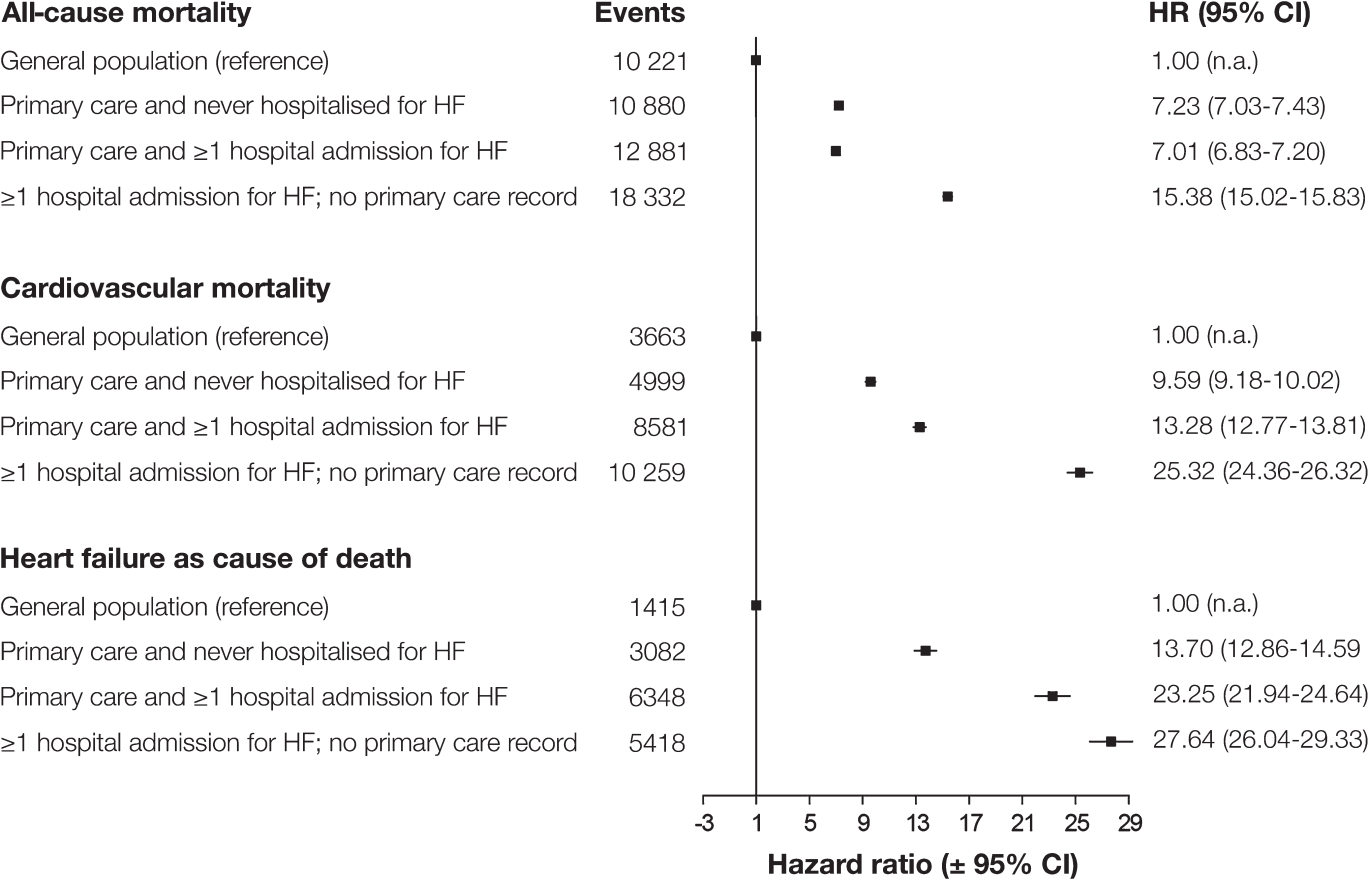
Cox proportional hazard models for association between electronic health record record for heart failure (HF) and 5‐year all‐cause, cardiovascular, and HF‐related mortality, stratified by HF recorded in primary care, acute HF hospital admissions, or both. CI, confidence interval; HR, hazard ratio.

We analysed 51 903 deaths over 206 055 person‐years follow‐up [median follow up 1.7 years (IQR 0.22–4.43)]. Out of those, 17 230 (33.1%) occurred within 3 months, and descriptive characteristics of patients dying within 3 months are listed in the Supplementary material online, *Table S7*. *Figure* 2 shows the Kaplan–Meier curves for 90‐day survival (*Figure* 2 *A–C*), and 5‐year survival in patients who survived the first 3 months following diagnosis (*Figure* 2 *D–F*). The crude 5‐year survival estimates for the above strata were 21.7% [95% confidence interval (CI) 21.1–22.2%], 43.9% (95% CI 43.2–44.6%), and 39.8% (95% CI 39.2–40.5%), respectively, compared with 88.1% (95% CI 87.9–88.3%) in the age‐ and sex‐matched general population (*Table* 2). Of note, all three groups had slightly lower survival in women than in men (*Table* 2). Corrected for age and sex, HF was strongly associated with mortality, with HRs for all‐cause mortality ranging from 7.01 (95% CI 6.83–7.20), 7.23 (95% CI 7.03–7.43), up to 15.38 (95% CI 15.02–15.83) for patients in primary care with acute HF hospitalization, primary care only, and patients hospitalized but no PC record, compared with the age‐ and sex‐matched general population (whose hazard was set as the reference) (*Figure* 3). With regard to risk factors associated with poor outcome, multivariable Cox proportional hazard models showed that age, concomitant COPD, and diabetes were amongst the strongest predictors of death (Supplementary material online, *Table S8*).

**Table 2:** Five‐year survival estimates with 95% confidence intervals in patients with heart failure recorded in primary care, hospital admissions, or both compared with the age‐ and sex‐matched general population

	Age‐ and sex‐matched general population	Source of HF record (HF population)		
		CPRD	CPRD and HES	HES
		Primary care record of HF but never hospitalized for HF	Primary care record of HF and hospitalized at least once for HF	Hospitalized for HF without primary care record of HF
No. of patients	77 857	23 547	23 681	30 629
Age < 55 years	99.7% (99.5–99.8%)	81.7% (79.1–84.3%)	69.7% (65.8–73.9%)	56.9% (53.7–60.3%)
Age 55–75 years	96.5% (96.3–96.7%)	62.1% (60.9–63.4%)	56.0% (54.7–57.3%)	40.0% (38.6–41.3%)
Age > 75 years	75.4% (74.9–75.8%)	32.7% (31.8–33.6%)	32.4% (31.6–33.2%)	13.2% (12.7–13.8%)
Men	88.7% (88.4–89.0%)	44.2% (43.2–45.3%)	41.3% (40.3–42.3%)	24.5% (23.6–25.4%)
Women	87.5% (87.2–87.8%)	43.6% (42.6–44.6%)	38.4% (37.4–39.3%)	19.2% (18.5–20.0%)
Total	88.1% (87.9–88.3%)	43.9% (43.2–44.6%)	39.8% (39.2–40.5%)	21.7% (21.1–22.2%)

CPRD, Clinical Practice Research Datalink; HES, Hospital Episodes Statistics; HF, heart failure.

## Discussion

We assessed EHR data of >2 million people for the presence of HF, within its real‐world context using three linked data sources: primary care (CPRD), hospital admissions for HF (HES), and the mortality registry (ONS). Among nearly 90 000 patients identified with HF, a quarter of all HF is recorded in primary care and these patients are never hospitalized for HF—such patients are largely excluded from trials, registries, and other studies—yet still had a high 5‐year mortality. Secondly, about a third of patients were given their first diagnosis of HF during an acute hospitalization but were not followed up after discharge in primary care. These patients had the worst 5‐year survival, and the worst medical management. Taken together, these findings support the wider use of linked EHR sources to facilitate a more consistent approach to identification, profiling, and treatment of HF patients in the real world to improve adherence to evidence‐based care and reduce mortality.

### Heart failure in primary care without heart failure‐related hospital admissions—the 26%

Importantly, we found that in the UK, approximately a quarter of all patients with HF in the general population were seen in primary care—and presumingly also in the outpatient secondary care setting—but were never acutely hospitalized for the disease. This is roughly similar to that reported in the only other investigation of the distribution of HF across different care settings.^6^ To our knowledge, this is the first study that assessed the prognostic burden of HF based on level of care. Here, we show that although acute hospitalizations for HF are well known for their negative impact on prognosis,^15^, ^16^, ^40^ unexpectedly 5‐year survival estimates were roughly comparable between primary care patients who had never been hospitalized for HF and those who had. As the natural course of HF commonly includes periods of destabilizations requiring a hospital admission, it is uncertain which factors other than HF hospital admissions are responsible for the low survival in these patients. Although under‐recording of hospital admissions in HES cannot be ruled out, patients in this group could also reflect end‐stage HF in which the decompensated patient was not referred to the hospital as part of end‐of‐life counselling by the GP, or that these patients died because of competing risks.

### Hospitalization for heart failure without a primary care record—the 34%

We found that ∼40% of all HF diagnoses in the general population were made as a result of an acute hospitalization. Importantly, our study shows (as has been described for other diseases, such as cancer^41^) that *de novo* hospital admissions were strongly associated with mortality compared with diagnoses that are formulated in primary care or in a hospital outpatient setting. While it is possible that some of these cases may have been fulminant, with no opportunity for diagnosis in the community, it is likely that in many cases the diagnosis during an acute admission was late and already associated with high levels of disease severity.

Besides disease severity, it stands to reason that at least part of their poor prognosis could be explained by their medical management, being the worst of all three groups with the lowest coverage of ACE inhibitors or ARBs, beta‐blockers, and MRAs (*Table* 1). Hence, the lack of a primary care record in these patients might be the result of a quality of care gap between primary and secondary care. Indeed, the importance of improved transition between hospital and ambulant settings was recently reinforced by Al‐Dumluji *et al.* showing that high quality discharge summaries were associated with a reduced number of readmissions.^42^

### Heart failure diagnosis at death—the 13%

With linkage to the national mortality register, this study advances previous reports from Sweden^6^ or Germany^7^ by quantifying HF‐specific mortality. Notably, we found that 13% of 89 554 patients allegedly died of HF but were not recorded as having had HF prior to their death in primary care, nor were they admitted to the hospital for HF (Supplementary material online, *Table S4*). Work to characterize these patients further showed that they were on average older, more frequently women, had higher levels of social deprivation, and showed fewer traditional cardiovascular risk factors. Although diagnosing HF as cause of death can be difficult, these findings suggest that there could be opportunities for improving screening, identification, and treatment of HF in these elderly patients, thereby potentially preventing HF‐attributable deaths.

### Limitations

The HF diagnoses used in this study were based on Read and/or ICD codes related to HF. In general, diagnoses recorded in CPRD or HES are sufficiently robust to permit their use for research purposes.^37^, ^43^ However, one limitation concerns the inability to validate a diagnosis of HF directly using a gold standard (i.e. confirm HF cases by expert panel review of clinical notes and diagnostic tests) as CALIBER data are anonymized to safeguard patient privacy. As in previous studies,^30^, ^31^, ^32^, ^39^, ^44^, ^45^ we first examined characteristics of patients identified by our EHR‐driven HF phenotype in terms of demographics, cardiovascular risk factors, and co‐morbidity. Overall, patient characteristics were similar to those observed in traditional HF registries^1^ as well as in EHR cohorts from other European countries,^6^, ^7^ which provides indirect evidence of the validity of the code lists used for the identification of patients with HF. Secondly, due to limitations inherent to routinely collected EHR data, low rates of medication use can be caused by under‐recording (i.e. prescriptions for HF medication not recorded by the GP in CPRD), misclassification of HF (i.e. no HF medication since patients do not have HF), true under‐treatment of HF patients, or a combination of the three. Overall, the impact of the above‐mentioned limitations are that, given an uncertain number of coding errors or misdiagnoses leading to false positives (i.e. patients recorded as having had HF but do not have HF in real life), our prognostic estimates might even be biased towards the conservative side.

### Clinical implications

Our findings have important clinical implications. First, the joint care in both primary and secondary care was associated with higher prescription rates of HF medication. This leaves two large groups of patients who could be potentially undertreated in current practice, being patients solely seen in ambulatory care (i.e. primary care and outpatient secondary care) and patients with a discharge diagnosis of HF in acute hospital admissions without a concurrent PC record. Higher levels of social deprivation in these two groups hint that certain HF patients are prone to suboptimal treatment in daily clinical care. Secondly, our findings underline the need for follow‐up after a discharge diagnosis of HF that should include a pro‐active diagnostic work‐up with joint effort in both PC and outpatient secondary care. With increasing evidence from the real world generated from programmes such as CALIBER or the ARNO database by Maggioni and co‐workers,^46^ novel high‐risk groups become apparent, and those described in this study are currently not reflected as high‐risk individuals in US or European guidelines.^47^, ^48^ Lastly, to date, almost all clinical trials have been enrolling patients with HF who have been hospitalized at least once. Here, we show that this choice impairs the generalizability towards a quarter of all HF patients in the general population who are never hospitalized. To increase external validity, we propose that future trials follow the BNP inclusion criterion as used in the PARADIGM‐HF trial involving sacubitril/valsartan, rather than include trial participants based on an acute HF‐related hospital admission.^49^, ^50^

## Conclusions

In the general population, one in four patients with newly diagnosed HF will not be hospitalized for HF within a median follow‐up of 1.7 years, yet they still have a dismal 5‐year prognosis. Patients admitted to hospital with HF but not known with HF in primary care have the worst management and survival. Mitigating the prognostic burden of HF requires greater consistency across primary and secondary care in the identification, profiling, and treatment of patients.

### Funding

S.K. is supported by a research fellowship programme of the non‐profit organization Genetic Cardiomyopathy PLN (http://hartspierziektepln.nl); A.D.S. is supported by a clinical research training fellowship from the Wellcome Trust (0938/30/Z/10/Z). F.A. is supported by a Dekker scholarship‐Junior Staff Member 2014 T001–Netherlands Heart Foundation and UCL Hospitals NIHR Biomedical Research Centre. The Farr Institute of Health Informatics Research at UCL Partners is funded based on awards from the Medical Research Council, Arthritis Research UK, British Heart Foundation, Cancer Research UK, Chief Scientist Office, Economic and Social Research Council, Engineering and Physical Sciences Research Council, National Institute for Health Research, National Institute for Social Care and Health Research, and Wellcome Trust (grant MR/K006584/1).

**Conflict of interest:** none declared.

## Supporting information


**Figure S1.** Flow chart of inclusion and exclusion CALIBER heart failure patients,
**Figure S2.** Diagram of patient groups based on electronic health record source.
**Figure S3.** Histogram of days to primary care record following index event of acute HF hospital admission recorded in Hospital Episode Statistics (HES).
**Figure S4.** Number of patients with hospital admissions and re‐admissions in Hospital Episode Statistics (HES) from 1 January 1997 to 26 March 2010.
**Table S1.** STROBE and RECORD checklist.
**Table S2.** Read codes used to identify heart failure in primary care (Clinical Practice Research Datalink).
**Table S3.** Read and OPCS (Office of Population Censuses and Surveys) codes used to identify supporting information for heart failure in primary care (Clinical Practice Research Datalink) and secondary care (OPCS).
**Table S4.** Characteristics of heart failure patients recorded in hospital admissions, alone or with a primary care record following the index hospitalization, or as cause of death but no other records of heart failure.
**Table S5.** Missing data on risk factors and patient characteristics in primary care for patients with heart failure recorded in primary care, hospital admissions, or death registry sources from 1 January 1997 to 26 March 2010.
**Table S6.** Recording of information supportive of a diagnosis in patients with heart failure recorded in primary care, hospital admissions, or mortality registry sources (n = 89 554).
**Table S7.** Recording of heart failure treatment and supportive information in primary care of patients with heart failure recorded in primary care, hospital admissions, or mortality registry, stratified by mortality status and timing of death.
**Table S8.** Association between patient characteristics and 5‐year all‐cause mortality after index heart failure diagnosis, adjusted for age and sex, and stratified by primary care practice.
