# Supplementary material for: Prognostic burden of heart failure recorded in primary care, acute hospital admissions, or both: a population‐based linked electronic health record cohort study in 2.1 million people
Source: Eur J Heart Fail. 2016 Dec 23;19(9):1119–27. doi: 10.1002/ejhf.709 (PMC5420446; doi:10.1002/ejhf.709)
Supplement: Supplementary file 1 — Figure S1. Flow chart of inclusion and exclusion CALIBER heart failure patients, Figure S2. Diagram of patient groups based on electronic health record source. Figure S3. Histogram of days to primary care record following index event of acute HF hospital admission recorded in Hospital Episode Statistics (HES). Figure S4. Number of patients with hospital admissions and re‐admissions in Hospital Episode Statistics (HES) from 1 January 1997 to 26 March 2010. Table S1. STROBE and RECORD checklist. Table S2. Read codes used to identify heart failure in primary care (Clinical Practice Research Datalink). Table S3. Read and OPCS (Office of Population Censuses and Surveys) codes used to identify supporting information for heart failure in primary care (Clinical Practice Research Datalink) and secondary care (OPCS). Table S4. Characteristics of heart failure patients recorded in hospital admissions, alone or with a primary care record following the index hospitalization, or as cause of death but no other records of heart failure. Table S5. Missing data on risk factors and patient characteristics in primary care for patients with heart failure recorded in primary care, hospital admissions, or death registry sources from 1 January 1997 to 26 March 2010. Table S6. Recording of information supportive of a diagnosis in patients with heart failure recorded in primary care, hospital admissions, or mortality registry sources (n = 89 554). Table S7. Recording of heart failure treatment and supportive information in primary care of patients with heart failure recorded in primary care, hospital admissions, or mortality registry, stratified by mortality status and timing of death. Table S8. Association between patient characteristics and 5‐year all‐cause mortality after index heart failure diagnosis, adjusted for age and sex, and stratified by primary care practice. [file EJHF-19-1119-s001.docx]

**Supplementary Table 1. STROBE and RECORD checklist**

**The RECORD statement – checklist of items, extended from the STROBE statement, that should be reported in observational studies using routinely collected health data.**

|  | **Item No.** | **STROBE items** | **Location in manuscript where items are reported** | **RECORD items** | **Location in manuscript where items are reported** |
| --- | --- | --- | --- | --- | --- |
| **Title and abstract** | | | | | |
|  | 1 | (a) Indicate the study’s design with a commonly used term in the title or the abstract (b) Provide in the abstract an informative and balanced summary of what was done and what was found | Title and abstract (p1-2) | RECORD 1.1: The type of data used should be specified in the title or abstract. When possible, the name of the databases used should be included.  RECORD 1.2: If applicable, the geographic region and timeframe within which the study took place should be reported in the title or abstract.  RECORD 1.3: If linkage between databases was conducted for the study, this should be clearly stated in the title or abstract. | 1.1 Listed in abstract under settings  1.2 listed in abstract under both setting and participants  1.3 listed in the title |
| **Introduction** | | | | | |
| Background rationale | 2 | Explain the scientific background and rationale for the investigation being reported | Introduction first and second paragraph |  |  |
| Objectives | 3 | State specific objectives, including any prespecified hypotheses | Introduction, third paragraph |  |  |
| **Methods** | | | | | |
| Study Design | 4 | Present key elements of study design early in the paper | Methods, study design and data sources |  |  |
| Setting | 5 | Describe the setting, locations, and relevant dates, including periods of recruitment, exposure, follow-up, and data collection | Methods, study design and data sources, study population |  |  |
| Participants | 6 | *(a) Cohort study* - Give the eligibility criteria, and the sources and methods of selection of participants. Describe methods of follow-up  *Case-control study* - Give the eligibility criteria, and the sources and methods of case ascertainment and control selection. Give the rationale for the choice of cases and controls  *Cross-sectional study* - Give the eligibility criteria, and the sources and methods of selection of participants  *(b) Cohort study* - For matched studies, give matching criteria and number of exposed and unexposed  *Case-control study* - For matched studies, give matching criteria and the number of controls per case | (a) methods section include eligibility criteria, sources, and method of selection. Follow up described as separate paragraph. | RECORD 6.1: The methods of study population selection (such as codes or algorithms used to identify subjects) should be listed in detail. If this is not possible, an explanation should be provided.  RECORD 6.2: Any validation studies of the codes or algorithms used to select the population should be referenced. If validation was conducted for this study and not published elsewhere, detailed methods and results should be provided.  RECORD 6.3: If the study involved linkage of databases, consider use of a flow diagram or other graphical display to demonstrate the data linkage process, including the number of individuals with linked data at each stage. | 6.1 reference to portal listed in methods section and supplementary table for new algorithms  6.2 listed in ‘clinical details of heart failure’  6.3 flow chart listed as supplementary figure. |
| Variables | 7 | Clearly define all outcomes, exposures, predictors, potential confounders, and effect modifiers. Give diagnostic criteria, if applicable. | See methods ‘baseline’ section | RECORD 7.1: A complete list of codes and algorithms used to classify exposures, outcomes, confounders, and effect modifiers should be provided. If these cannot be reported, an explanation should be provided. | Referenced to CALIBER portal in method section. |
| Data sources/ measurement | 8 | For each variable of interest, give sources of data and details of methods of assessment (measurement).  Describe comparability of assessment methods if there is more than one group | See methods ‘baseline’ section |  |  |
| Bias | 9 | Describe any efforts to address potential sources of bias | See methods ‘statistical analyses’ section |  |  |
| Study size | 10 | Explain how the study size was arrived at | See methods ‘study design’ section |  |  |
| Quantitative variables | 11 | Explain how quantitative variables were handled in the analyses. If applicable, describe which groupings were chosen, and why | See methods ‘statistical analyses’ |  |  |
| Statistical methods | 12 | (a) Describe all statistical methods, including those used to control for confounding  (b) Describe any methods used to examine subgroups and interactions  (c) Explain how missing data were addressed  (d) *Cohort study* - If applicable, explain how loss to follow-up was addressed  *Case-control study* - If applicable, explain how matching of cases and controls was addressed  *Cross-sectional study* - If applicable, describe analytical methods taking account of sampling strategy  (e) Describe any sensitivity analyses | See methods ‘statistical analyses’ |  |  |
| Data access and cleaning methods |  | .. |  | RECORD 12.1: Authors should describe the extent to which the investigators had access to the database population used to create the study population.  RECORD 12.2: Authors should provide information on the data cleaning methods used in the study. | 12.1 see methods ‘study design and data sources’ section  12.2 definitions used are described in methods section |
| Linkage |  | .. |  | RECORD 12.3: State whether the study included person-level, institutional-level, or other data linkage across two or more databases. The methods of linkage and methods of linkage quality evaluation should be provided. | Referenced to CALIBER portal, which includes extensive information. Referenced to paper explaining the CALIBER program (S Denaxas et al, Int J Epidemiology) |
| **Results** | | | | | |
| Participants | 13 | (a) Report the numbers of individuals at each stage of the study (*e.g.*, numbers potentially eligible, examined for eligibility, confirmed eligible, included in the study, completing follow-up, and analysed)  (b) Give reasons for non-participation at each stage.  (c) Consider use of a flow diagram | Results ‘distribution of heart failure…’ section  Flow chart mentioned in supplementary information | RECORD 13.1: Describe in detail the selection of the persons included in the study (*i.e.,* study population selection) including filtering based on data quality, data availability and linkage. The selection of included persons can be described in the text and/or by means of the study flow diagram. | 13.1 Results ‘distribution of heart failure…’ section  Flow chart mentioned in supplementary information |
| Descriptive data | 14 | (a) Give characteristics of study participants (*e.g.*, demographic, clinical, social) and information on exposures and potential confounders  (b) Indicate the number of participants with missing data for each variable of interest  (c) *Cohort study* - summarise follow-up time (*e.g.*, average and total amount) | See main table 1  B: listed in supplementary files  C: listed in main text of results |  |  |
| Outcome data | 15 | *Cohort study* - Report numbers of outcome events or summary measures over time  *Case-control study* - Report numbers in each exposure category, or summary measures of exposure  *Cross-sectional study* - Report numbers of outcome events or summary measures | Listed in main text of results |  |  |
| Main results | 16 | (a) Give unadjusted estimates and, if applicable, confounder-adjusted estimates and their precision (e.g., 95% confidence interval). Make clear which confounders were adjusted for and why they were included  (b) Report category boundaries when continuous variables were categorized  (c) If relevant, consider translating estimates of relative risk into absolute risk for a meaningful time period | See main table 2, main figure 1 to 3 |  |  |
| Other analyses | 17 | Report other analyses done—e.g., analyses of subgroups and interactions, and sensitivity analyses | Correction in Cox models listed in methods as well as main text. |  |  |
| **Discussion** | | | | | |
| Key results | 18 | Summarise key results with reference to study objectives | First paragraph of discussion section |  |  |
| Limitations | 19 | Discuss limitations of the study, taking into account sources of potential bias or imprecision. Discuss both direction and magnitude of any potential bias | See discussion, limitation section | RECORD 19.1: Discuss the implications of using data that were not created or collected to answer the specific research question(s). Include discussion of misclassification bias, unmeasured confounding, missing data, and changing eligibility over time, as they pertain to the study being reported. | See discussion, limitation section. |
| Interpretation | 20 | Give a cautious overall interpretation of results considering objectives, limitations, multiplicity of analyses, results from similar studies, and other relevant evidence | See discussion |  |  |
| Generalisability | 21 | Discuss the generalisability (external validity) of the study results | See discussion, clinical implications |  |  |
| **Other Information** | | | | | |
| Funding | 22 | Give the source of funding and the role of the funders for the present study and, if applicable, for the original study on which the present article is based | See funding |  |  |
| Accessibility of protocol, raw data, and programming code |  | .. | See data sharing agreement | RECORD 22.1: Authors should provide information on how to access any supplemental information such as the study protocol, raw data, or programming code. | See data sharing agreement |

*Reference: Benchimol EI, Smeeth L, Guttmann A, Harron K, Moher D, Petersen I, Sørensen HT, von Elm E, Langan SM, the RECORD Working Committee. The REporting of studies Conducted using Observational Routinely-collected health Data (RECORD) Statement. *PLoS Medicine* 2015; in press.

*Checklist is protected under Creative Commons Attribution ([CC BY](http://creativecommons.org/licenses/by/4.0/)) license.

**Supplementary Table 2. Read codes used to identify heart failure in primary care (Clinical Practice Research Datalink).**

| **Clinical code** | **Clinical term** |
| --- | --- |
| 1736.00 | Paroxysmal nocturnal dyspnoea |
| 1J60.00 | Suspected heart failure |
| 23E1.00 | O/E - pulmonary oedema |
| 388D.00 | New York Heart Assoc classification heart failure symptoms |
| 662T.00 | Congestive heart failure monitoring |
| 662f.00 | New York Heart Association classification - class I |
| 662g.00 | New York Heart Association classification - class II |
| 662h.00 | New York Heart Association classification - class III |
| 662i.00 | New York Heart Association classification - class IV |
| 679X.00 | Heart failure education |
| 8CL3.00 | Heart failure care plan discussed with patient |
| 8HBE.00 | Heart failure follow-up |
| 8HHz.00 | Referral to heart failure exercise programme |
| 8Hg8.00 | Discharge from practice nurse heart failure clinic |
| 8Hk0.00 | Referred to heart failure education group |
| 9N0k.00 | Seen in heart failure clinic |
| 9N2p.00 | Seen by community heart failure nurse |
| 9N4s.00 | Did not attend practice nurse heart failure clinic |
| 9N4w.00 | Did not attend heart failure clinic |
| 9N6T.00 | Referred by heart failure nurse specialist |
| 9On..00 | Left ventricular dysfunction monitoring administration |
| 9On0.00 | Left ventricular dysfunction monitoring first letter |
| 9On1.00 | Left ventricular dysfunction monitoring second letter |
| 9On2.00 | Left ventricular dysfunction monitoring third letter |
| 9On3.00 | Left ventricular dysfunction monitoring verbal invite |
| 9On4.00 | Left ventricular dysfunction monitoring telephone invite |
| 9Or..00 | Heart failure monitoring administration |
| 9Or1.00 | Heart failure monitoring telephone invite |
| 9Or2.00 | Heart failure monitoring verbal invite |
| 9Or3.00 | Heart failure monitoring first letter |
| 9Or4.00 | Heart failure monitoring second letter |
| 9Or5.00 | Heart failure monitoring third letter |
| 9h1..00 | Exception reporting: LVD quality indicators |
| 9h11.00 | Excepted from LVD quality indicators: Patient unsuitable |
| 9h12.00 | Excepted from LVD quality indicators: Informed dissent |
| 9hH..00 | Exception reporting: heart failure quality indicators |
| 9hH0.00 | Excepted heart failure quality indicators: Patient unsuitabl |
| 9hH1.00 | Excepted heart failure quality indicators: Informed dissent |
| G581.12 | Pulmonary oedema - acute |
| G58z.11 | Weak heart |
| H54..00 | Pulmonary congestion and hypostasis |
| H541.00 | Pulmonary congestion |
| H541000 | Chronic pulmonary oedema |
| H541z00 | Pulmonary oedema NOS |
| H54z.00 | Pulmonary congestion and hypostasis NOS |
| H584.00 | Acute pulmonary oedema unspecified |
| H584z00 | Acute pulmonary oedema NOS |
| ZRad.00 | New York Heart Assoc classification heart failure symptoms |
| G580400 | Congestive heart failure due to valvular disease |
| G210.00 | Malignant hypertensive heart disease |
| G210000 | Malignant hypertensive heart disease without CCF |
| G210100 | Malignant hypertensive heart disease with CCF |
| G211100 | Benign hypertensive heart disease with CCF |
| G21z100 | Hypertensive heart disease NOS with CCF |
| G230.00 | Malignant hypertensive heart and renal disease |
| G232.00 | Hypertensive heart&renal dis wth (congestive) heart failure |
| G234.00 | Hyperten heart&renal dis+both(congestv)heart and renal fail |
| G1yz100 | Rheumatic left ventricular failure |
| 1O1..00 | Heart failure confirmed |
| 662W.00 | Heart failure annual review |
| 662p.00 | Heart failure 6 month review |
| 8B29.00 | Cardiac failure therapy |
| 8H2S.00 | Admit heart failure emergency |
| 9Or0.00 | Heart failure review completed |
| G400.00 | Acute cor pulmonale |
| G41z.11 | Chronic cor pulmonale |
| G554000 | Congestive cardiomyopathy |
| G554011 | Congestive obstructive cardiomyopathy |
| G58..00 | Heart failure |
| G58..11 | Cardiac failure |
| G580.00 | Congestive heart failure |
| G580.11 | Congestive cardiac failure |
| G580.12 | Right heart failure |
| G580.13 | Right ventricular failure |
| G580.14 | Biventricular failure |
| G580000 | Acute congestive heart failure |
| G580100 | Chronic congestive heart failure |
| G580200 | Decompensated cardiac failure |
| G580300 | Compensated cardiac failure |
| G581.00 | Left ventricular failure |
| G581.11 | Asthma - cardiac |
| G581.13 | Impaired left ventricular function |
| G581000 | Acute left ventricular failure |
| G582.00 | Acute heart failure |
| G58z.00 | Heart failure NOS |
| G58z.12 | Cardiac failure NOS |
| G5yy900 | Left ventricular systolic dysfunction |
| G5yyA00 | Left ventricular diastolic dysfunction |
| R2y1000 | [D]Cardiorespiratory failure |

**Supplementary Table 3. Read and OPCS (Office of Population Censuses and Surveys) codes used to identify supporting information for heart failure in primary care (Clinical Practice Research Datalink) and secondary care (OPCS).**

| **Signs and Symptoms** | |
| --- | --- |
| **Read code** | **Read term** |
| R095z00 | [D]Ascites NOS |
| 25OZ.00 | O/E - ascites NOS |
| R2y4.00 | [D]Cachexia |
| R2y4z00 | [D]Cachexia NOS |
| 173D.00 | Nocturnal dyspnoea |
| R060A00 | [D]Dyspnoea |
| 173C.11 | Dyspnoea on exertion |
| 173..12 | Dyspnoea - symptom |
| 2322.00 | O/E - dyspnoea |
| R007100 | [D]Fatigue |
| 168..11 | Fatigue - symptom |
| Eu46011 | [X]Fatigue syndrome |
| R007.00 | [D]Malaise and fatigue |
| 1682 | Fatigue |
| G581.11 | Asthma - cardiac |
| 662g.00 | New York Heart Association classification - class II |
| H541.00 | Pulmonary congestion |
| 662f.00 | New York Heart Association classification - class I |
| 662h.00 | New York Heart Association classification - class III |
| H541000 | Chronic pulmonary oedema |
| ZRad.00 | New York Heart Assoc classification heart failure symptoms |
| H54..00 | Pulmonary congestion and hypostasis |
| 68B6.00 | Heart failure screen |
| 388D.00 | New York Heart Assoc classification heart failure symptoms |
| 662i.00 | New York Heart Association classification - class IV |
| 23E1.00 | O/E - pulmonary oedema |
| H584z00 | Acute pulmonary oedema NOS |
| H584.00 | Acute pulmonary oedema unspecified |
| H54z.00 | Pulmonary congestion and hypostasis NOS |
| 1736.00 | Paroxysmal nocturnal dyspnoea |
| H541z00 | Pulmonary oedema NOS |
| R007300 | [D]Lethargy |
| 1684.00 | Malaise/lethargy |
| 168..12 | Lethargy - symptom |
| R030000 | [D]Appetite loss |
| 1612.12 | Loss of appetite - symptom |
| 1612.00 | Appetite loss - anorexia |
| 22C2.11 | O/E - ankle oedema |
| 183..00 | Oedema |
| R023.00 | [D]Oedema |
| 23E1.00 | O/E - pulmonary oedema |
| 183..11 | Oedema - symptom |
| 22C4.11 | O/E - leg oedema |
| 22C3.11 | O/E - foot oedema |
| R060200 | [D]Orthopnoea |
| 2323.00 | O/E - orthopnoea |
| 1735.11 | Orthopnoea symptom |
| Hyu7000 | [X]Pleural effusion in conditions classified elsewhere |
| RIGHT V | RIGHT VENTRICULAR HEAVE |
| R060800 | [D]Shortness of breath |
| 1739 | Shortness of breath |
| 173..13 | Shortness of breath symptom |
| 388H.00 | CLASP shortness of breath score |
| ZR3Q.00 | CLASP shortness of breath score |
| H584.00 | Acute pulmonary oedema unspecified |
| 22C3.00 | O/E - oedema of feet |
| H584z00 | Acute pulmonary oedema NOS |
| H541z00 | Pulmonary oedema NOS |
| 1837 | Pitting oedema |
| 22C7.00 | O/E - sacral oedema |
| 22C..00 | O/E - oedema |
| R023400 | [D]Peripheral oedema |
| R023z00 | [D]Oedema NOS |
| R023300 | [D]Oedema, localized |
| R023z11 | [D]Dependent oedema |
| 22C4.00 | O/E - oedema of legs |
| 22C5.11 | O/E - thigh oedema |
| R023000 | [D]Oedema, generalized |
| 22C2.00 | O/E - oedema of ankles |
| 8E95.00 | Reduction of oedema |
| 1838 | Sacral oedema |
| H541000 | Chronic pulmonary oedema |
| 22CZ.00 | O/E - oedema NOS |
| 183Z.00 | Oedema NOS |
| H584000 | Postoperative pulmonary oedema |
| G581.12 | Pulmonary oedema - acute |
| H584.11 | Acute oedema of lung, unspecified |
| 22C5.00 | O/E - oedema of thighs |
| C366100 | Fluid retention |
| R095.00 | [D]Ascites |
| 25O..00 | O/E - ascites |
| H51yz00 | Other pleural effusion |
| 7H2B000 | Paracentesis abdominis for ascites |
| 23D4.00 | O/E - fine crepitations |
| 23D3.00 | O/E - coarse crepitations |
| 24B9.00 | O/E - third heart sound |
| H51y.00 | Other pleural effusion excluding mention of tuberculosis |
| 7H2B200 | Drainage of ascites NEC |
| R095z00 | [D]Ascites NOS |
| 25O4.00 | O/E -ascites-shifting dullness |
| Hyu7000 | [X]Pleural effusion in conditions classified elsewhere |
| DISPLAC | DISPLACED APEX BEAT |
| RIGHT V | RIGHT VENTRICULAR HEAVE |
| 25OZ.00 | O/E - ascites NOS |
| DISPLAC | DISPLACED APEX BEAT |
| 25O3.00 | O/E-ascites-fluid thrill shown |
| RIGHT V | RIGHT VENTRICULAR HEAVE |
| 23D..12 | O/E - crepitations |
| RIGHT V | RIGHT VENTRICULAR HEAVE |
| H51z.00 | Pleural effusion NOS |
| H51zz00 | Pleural effusion NOS |
| RIGHT V | RIGHT VENTRICULAR HEAVE |
| N247013 | Swollen legs |
| 183..12 | Swelling - oedema - symptom |
| 1832,11 | Ankle swelling symptom |
| 1833,11 | Leg swelling symptom |
| N247012 | Swollen lower leg |
| 22C..11 | O/E - swelling - oedema |
| 1832 | Ankle swelling |
| 16J7.00 | Swollen foot |
| 1833 | Leg swelling |
| ZR3R.00 | Cardiovasc Limits and Symptoms Profile ankle swelling score |
| ZR3R.11 | CLASP ankle swelling score |
| G570000 | Paroxysmal atrial tachycardia |
| G572z00 | Paroxysmal tachycardia NOS |
| R050.00 | [D]Tachycardia, unspecified |
| G57y900 | Supraventricular tachycardia NOS |
| G570100 | Paroxysmal atrioventricular tachycardia |
| G572.00 | Paroxysmal tachycardia unspecified |
| 2426.00 | O/E - pulse rate tachycardia |
| G570300 | Paroxysmal nodal tachycardia |
| 14AP.00 | History of ventricular tachycardia |
| G571.00 | Paroxysmal ventricular tachycardia |
| G570z00 | Paroxysmal supraventricular tachycardia NOS |
| 3282.00 | ECG: ventricular tachycardia |
| G570.00 | Paroxysmal supraventricular tachycardia |
| G570200 | Paroxysmal junctional tachycardia |
| G57y700 | Sinus tachycardia |
| 2426.11 | O/E - tachycardia |
| G571.11 | Ventricular tachycardia |
| H584z00 | Acute pulmonary oedema NOS |
| H584.00 | Acute pulmonary oedema unspecified |
| H541000 | Chronic pulmonary oedema |
| ZRad.00 | New York Heart Assoc classification heart failure symptoms |
| 388D.00 | New York Heart Assoc classification heart failure symptoms |
| 662f.00 | New York Heart Association classification - class I |
| 662g.00 | New York Heart Association classification - class II |
| 662h.00 | New York Heart Association classification - class III |
| 662i.00 | New York Heart Association classification - class IV |
| 23E1.00 | O/E - pulmonary oedema |
| 1736.00 | Paroxysmal nocturnal dyspnoea |
| H541.00 | Pulmonary congestion |
| H54..00 | Pulmonary congestion and hypostasis |
| H54z.00 | Pulmonary congestion and hypostasis NOS |
| G581.12 | Pulmonary oedema - acute |
| H541z00 | Pulmonary oedema NOS |

| **Referral to specialised heart failure care** | |
| --- | --- |
| **Read code** | **Read term** |
| 8HTL.00 | Referral to heart failure clinic |
| 8HHz.00 | Referral to heart failure exercise programme |
| 8HHb.00 | Referral to heart failure nurse |
| 9N6T.00 | Referred by heart failure nurse specialist |
| 8Hk0.00 | Referred to heart failure education group |
| 8HQ7.00 | Referral for echocardiography |

| **Cardiac care** | |
| --- | --- |
| **Read code** | **Read term** |
| 662p.00 | Heart failure 6 month review |
| 662T.00 | Congestive heart failure monitoring |
| 662W.00 | Heart failure annual review |
| 679X.00 | Heart failure education |
| 67D4.00 | Heart failure information given to patient |
| 8B29.00 | Cardiac failure therapy |
| 8CL3.00 | Heart failure care plan discussed with patient |
| 8H11.00 | Admit to cardiac ITU |
| 8H2Q.00 | Admit cardiology emergency |
| 8HBE.00 | Heart failure follow-up |
| 8Hg8.00 | Discharge from practice nurse heart failure clinic |
| 8HK4.00 | Cardiology D.V. requested |
| 8HL4.00 | Cardiology D.V. done |
| 9N0k.00 | Seen in heart failure clinic |
| 9N0q.00 | Seen in cardiothoracic surgery clinic |
| 9N1P.00 | Seen in cardiac clinic |
| 9N1P.11 | Seen in cardiology clinic |
| 9N2p.00 | Seen by community heart failure nurse |
| 9N2x.00 | Seen by private cardiologist |
| 9N6T.00 | Referred by heart failure nurse specialist |
| 9N6V.00 | Referred by cardiologist |
| 9N6W.00 | Referred by cardiology outpatients department |
| 9NJK.00 | In-house cardiology first appointment |
| 9NJL.00 | In-house cardiology follow-up appointment |
| 9NJM.00 | In-house cardiology discharged from care |
| 9Nl1.00 | Seen by general practitioner special interest in cardiology |
| 9NM2.00 | Attending cardiology clinic |
| 9Oa..00 | Cardiovascular clinic |
| 9On..00 | Left ventricular dysfunction monitoring administration |
| 9On0.00 | Left ventricular dysfunction monitoring first letter |
| 9On1.00 | Left ventricular dysfunction monitoring second letter |
| 9On2.00 | Left ventricular dysfunction monitoring third letter |
| 9On3.00 | Left ventricular dysfunction monitoring verbal invite |
| 9On4.00 | Left ventricular dysfunction monitoring telephone invite |
| 9Or..00 | Heart failure monitoring administration |
| 9Or0.00 | Heart failure review completed |
| 9Or1.00 | Heart failure monitoring telephone invite |
| 9Or2.00 | Heart failure monitoring verbal invite |
| 9Or3.00 | Heart failure monitoring first letter |
| 9Or4.00 | Heart failure monitoring second letter |
| 9Or5.00 | Heart failure monitoring third letter |
| ZL18300 | Under care of cardiologist |
| ZL1G200 | Under care of cardiothoracic surgeon |
| ZL1G400 | Under care of cardiac surgeon |
| ZL9A300 | Seen by cardiologist |
| ZLD3300 | Discharge by cardiologist |
| ZLE6300 | Discharge from cardiology service |

| **Cardiac Imaging** | |
| --- | --- |
| **Read code** | **Read term** |
| 1I5..00 | No evidence of left ventricular diastolic dysfunction** |
| 5853000 | Echocardiogram normal |
| 585R.00 | Echocardiogram normal |
| G5y3411 | Left ventricular hypertrophy |
| R132000 | [D]Echocardiogram abnormal |
| 585f.00 | Echocardiogram shows left ventricular systolic dysfunction |
| G5yy900 | Left ventricular systolic dysfunction |
| 32BA.00 | Impaired left ventricular function |
| G581.13 | Impaired left ventricular function |
| 585f.00 | Echocardiogram shows left ventricular systolic dysfunction |
| 1I3..00 | No evidence of left ventricular systolic dysfunction |
| 585g.00 | Echocardiogram shows left ventricular diastolic dysfunction |
| 585k.00 | Echocardiogram shows normal left ventricular function |
| G5yyA00 | Left ventricular diastolic dysfunction |
| 585k.00 | Echocardiogram shows normal left ventricular function |
| 585g.00 | Echocardiogram shows left ventricular diastolic dysfunction |
| 33BB.00 | Left ventricular ejection fraction* |
| 56F1.00 | Echocardiogram declined |
| 7P0H500 | Fetal echocardiography |
| R143200 | [D]Ballistocardiogram abnormal |
| R143300 | [D]Phonocardiogram abnormal |
| R132200 | [D]Ultrasound cardiogram abnormal |
| R143400 | [D]Vectorcardiogram abnormal |
| 5C20.00 | Echocardiogram equivocal |
| 33BD.00 | Echocardiogram requested |
| 7P0H300 | Epicardial echocardiography |
| 8HQ7.00 | Referral for echocardiography |
| 7935500 | Transluminal intracardiac echocardiography |
| **OPCS code** | **OPCS procedure** |
| U20 | Diagnostic echocardiography |
| U20.8 | Other specified diagnostic Echocardiography |
| U20.5 | Stress echocardiography |
| U20.2 | Transoesophageal echocardiography |
| U20.1 | Transthoracic echocardiography |
| U20.9 | Unspecified diagnostic Echocardiography |

| **Supplementary Table 4.** **Characteristics of heart failure patients recorded in hospital admissions, alone or with a primary care record following the index hospitalisation, or as cause of death but no other records of HF.** | | | |
| --- | --- | --- | --- |
|  | **CPRD and HES** | | **ONS** |
| **Patient group** | **Primary care record of HF first and at least once hospitalised for HF thereafter** | **Hospitalised for HF with concurrent primary care record of HF after discharge** | **HF as cause of death but no primary care and/or hospital record** |
| Number of patients | 14 694 | 8987 | 11 697 |
| **Patient characteristics** |  |  |  |
| Median age [IQR] in years | 80.0 [72.4 – 86.3] | 80.3 [72.6 – 86.5] | 82.9 [74.1 – 89.4] |
| Women (%) | 7549 (51.3%) | 4512 (50.2%) | 6236 (53.3%) |
| Most deprived fifth (%)* | 2673 (18.2%) | 1485 (16.5%) | 2678 (22.9%) |
| Current Smoking^†^ (%) | 2024 (15.3%) | 1371 (16.6%) | 383 (12.8%) |
| Ex-smoker^†^ (%) | 4264 (32.2%) | 2748 (33.1%) | 888 (29.8%) |
| Never smoked^†^ (%) | 6937 (52.4%) | 4162 (50.3%) | 1721 (57.5%) |
| Body Mass Index^†^ mean and SD | 27.0 ± 5.8 | 27.2 ± 6.1 | 26.3 ± 6.2 |
| Systolic blood pressure in mmHg mean and SD^†^ | 140 ± 23 | 138 ± 24 | 132 ± 24 |
| Serum creatinine in µmol/L^†^ | 114 ± 60 | 119 ± 62 | 135 ± 80 |
| Haemoglobin in mmol/L^†^ | 12.9 ± 1.9 | 12.7 ± 1.9 | 12.3 ± 2.0 |
| **Comorbidity** |  |  |  |
| Hypertension^§^ (%) | 11800 (80.3%) | 7703 (85.7%) | 3586 (30.7%) |
| Diabetes mellitus^§^ (%) | 2110 (14.4%) | 1682 (18.7%) | 685 (5.9%) |
| Atrial Fibrillation^§^ (%) | 5670 (38.6%) | 4378 (48.7%) | 1835 (15.7%) |
| Ischaemic heart disease^§^ (%) | 7466 (50.8%) | 5358 (59.6%) | 1693 (14.5%) |
| Myocardial Infarction^§^ (%) | 3974 (27.0%) | 3106 (34.5%) | 1138 (9.7%) |
| Stroke^§^ (%) | 715 (4.8%) | 454 (5.1%) | 216 (1.8%) |
| COPD^§^ (%) | 3077 (20.9%) | 2237 (24.8%) | 969 (8.2%) |
| Depression^§^ (%) | 3041 (20.7%) | 1829 (20.3%) | 868 (7.4%) |
| Cancer^§^ (%) | 3142 (21.3%) | 1838 (20.5%) | 823 (7.0%) |
| **Heart failure medication** |  |  |  |
| Loop diuretics‡ (%) | 9695 (66.0%) | 7746 (86.2%) | 2652 (22.7%) |
| ACEi/ARB‡ (%) | 8034 (54.7%) | 6324 (70.3%) | 1823 (15.6%) |
| Betablockers | 5478 (37.2%) | 3320 (36.9%) | 541 (4.6%) |
| HF betablockers‡\|\|(%) | 2290 (15.6%) | 2281 (25.4%) | 369 (3.2%) |
| Mineralocorticoid receptor antagonists‡ (%) | 1949 (13.2%) | 2289 (25.5%) | 711 (6.1%) |
| *assessed by index of multiple deprivation † measurement closest to and within 6 months before or on the date of heart failure diagnosis. § denotes prior medical history of given comorbidity. ‡ denotes present or prescribed ± 6 months of index date for heart failure diagnosis. \|\| HF betablockers include metoprolol, carvedilol, or bisoprolol. | | | |

| **Supplementary Table 5. Missing data on risk factors and patient characteristics in primary care for patients with heart failure recorded in primary care, hospital admissions or death registry sources from 1 January 1997 to 26 March 2010** | | | | |
| --- | --- | --- | --- | --- |
|  | **Source of heart failure record** | | | |
|  | **CPRD** | **CPRD and HES** | **HES** | **ONS** |
| **Patient group** | **Primary care diagnosis of HF only** | **Primary care diagnosis of HF and at least one HF hospitalisation ever** | **Hospitalised for HF without primary care diagnosis of HF ever** | **HF as cause of death but no primary care and/or hospital record** |
| Number of patients | 23 547 | 23 681 | 30 629 | 11 697 |
| Age (% missing) | 0 (0%) | 0 (0%) | 0 (0%) | 0 (0%) |
| Gender (% missing) | 0 (0%) | 0 (0%) | 0 (0%) | 0 (0%) |
| Social deprivation (% missing)* | 0 (0%) | 0 (0%) | 0 (0%) | 0 (0%) |
| Smoking status^†^ (% missing) | 2592 (11.0%) | 1787 (7.5%) | 4176 (13.6%) | 8705 (74.4%) |
| Body Mass Index^†^ (% missing) | 4352 (18.5%) | 3653 (15.4%) | 6427 (21.0%) | 9107 (77.9%) |
| Systolic blood pressure in mmHg^†^  (% missing) | 619 (2.6%) | 320 (1.4%) | 1583 (5.2%) | 7759 (66.3%) |
| Serum creatinine in µmol/L^†^ | 4793 (20.4%) | 2941 (12.4%) | 7950 (26.0%) | 8607 (73.6%) |
| CPRD=Clinical Practice Research Datalink; HES=Hospital Episode Statistics; ONS=Office for National Statistics  *assessed by index of multiple deprivation  † measurement closest to date of heart failure diagnosis from all measurements recorded before diagnosis or within six months after diagnosis. | | | | |

| **Supplementary Table 6.** **Recording of information supportive of a diagnosis in patients with heart failure recorded in primary care, hospital admissions or mortality registry sources (n = 89 554)** | | | | |  |
| --- | --- | --- | --- | --- | --- |
|  | **Source of heart failure record** | | | |  |
|  | **CPRD** | **CPRD and HES** | **HES** | **ONS** |  |
| **Patient group** | **Primary care diagnosis of HF only** | **Primary care diagnosis of HF and at least once hospitalised for HF** | **Hospitalised for HF without primary care diagnosis of HF** | **HF as cause of death but no primary care and/or hospital record** |  |
| Number of patients | 23 547 | 23 681 | 30 629 | 11 697 |  |
| **Recorded supporting information** | |  |  |  |  |
| Signs and symptoms‡ (%) | | 12 377 (52.6%) | 16 197 (68.4%) | 7398 (24.1%) | 1036 (8.9%) |
| (NT-pro)BNP measured‡ (%) | 643 (2.7%) | 412 (1.7%) | 170 (0.6%) | 63 (0.5%) |  |
| (NT-pro)BNP elevated‡ (%) | 442 (1.9%) | 322 (1.4%) | 127 (0.4%) | 44 (0.4%) |  |
| Cardiac imaging ‡ (%) | 2778 (11.8%) | 4319 (18.2%) | 3360 (11.0%) | 85 (0.7%) |  |
| Cardiac care‡ (%) | 3251 (13.8%) | 4060 (17.1%) | 2480 (8.0%) | 0 (0.0%) |  |
| Referral to HF specialist‡ (%) | 489 (2.1%) | 509 (2.1%) | 68 (0.2%) | 0 (0.0%) |  |
| CPRD=Clinical Practice Research Datalink; HES=Hospital Episode Statistics; ONS=Office for National Statistics  . ‡ denotes present or prescribed ± 6 months of index date for heart failure diagnosis. | | | | |  |


| **Supplementary Table 7.** **Recording of heart failure treatment and supportive information in primary care of patients with heart failure recorded in primary care, hospital admissions or mortality registry, stratified by mortality status and timing of death.** | | | |
| --- | --- | --- | --- |
| **Patient group** | **Patients who died**  **<3 months following HF diagnosis** | **Patients who died**  **>3 months following HF diagnosis** | **All patients who survived** |
| Number of patients | 17 239 | 34 050 | 27 565 |
| Median age [IQR] in years | 83.5 [76.4 – 89.1] | 81.7 [75.3 – 87.3] | 74.3 [65.1 – 81.7] |
| Women (%) | 9526 (55.3%) | 18 015 (52.9%) | 13 095 (47.5%) |
| Most deprived fifth (%) | 3369 (19.5%) | 6501 (19.1%) | 5427 (19.7%) |
| Median follow up [IQR] in years | 0.03 [0.0 – 0.1] | 2.4 [1.1 – 4.7] | 3.4 [1.8 – 6.5] |
| Primary care record (CPRD) (%) | 5826 (33.8%) | 16 506 (71.3%) | 16 506 (59.8%) |
| HF hospitalisation record (HES) (%) | 12 381 (71.8%) | 24 173 (70.9%) | 17 747 (64.4%) |
| Current Smoking^†^ (%) | 2311 (16.8%) | 4475 (15.0%) | 4611 (17.5%) |
| Ex-smoker^†^ (%) | 3697 (26.8%) | 9021 (30.3%) | 9412 (35.7%) |
| Never smoked^†^ (%) | 7782 (56.4%) | 16 250 (54.7%) | 12 335 (46.8%) |
| Body Mass Index^†^ mean and SD | 25.9 ± 5.7 | 26.5 ± 5.6 | 28.4 ± 6.0 |
| Systolic blood pressure in mmHg  mean and SD^†^ | 139 ± 23 | 142 ± 24 | 138 ± 22 |
| Serum creatinine in µmol/L mean and SD^†^ | 118.9 ± 74.4 | 118.8 ± 62.6 | 104.7 ± 77.3 |
| Ischemic heart disease^§^ (%) | 6959 (40.3%) | 17 621 (51.8%) | 14 598 (52.9%) |
| Myocardial Infarction^§^ (%) | 4103 (23.8%) | 9376 (27.5%) | 7819 (28.3%) |
| Hypertension^§^ (%) | 12 993 (75.3%) | 28 127 (82.6%) | 21 669 (78.6%) |
| Diabetes mellitus^§^ (%) | 2684 (15.6%) | 5005 (14.7%) | 3854 (13.9%) |
| Atrial Fibrillation^§^ (%) | 5934 (34.4%) | 13 950 (40.9%) | 10 006 (36.3%) |
| Stroke^§^ (%) | 1025 (5.9%) | 1651 (4.8%) | 1026 (3.7%) |
| COPD^§^ (%) | 3456 (20.0%) | 8089 (23.7%) | 4524 (16.4%) |
| Depression^§^ (%) | 3310 (19.2%) | 6905 (20.2%) | 5834 (21.1%) |
| Cancer^§^ (%) | 4561 (26.4%) | 7569 (22.2%) | 4358 (15.8%) |
| Loop diuretics^‡^ (%) | 7826 (45.3%) | 27 199 (79.8%) | 16 298 (59.1%) |
| ACEi/ARB^‡^ (%) | 4996 (28.9%) | 19 609 (57.5%) | 17 494 (63.4%) |
| Betablockers | 3142 (18.2%) | 8959 (25.2%) | 11 647 (42.3%) |
| Cardioselective betablockers^‡\|\|^ (%) | 800 (4.6%) | 3997 (11.7%) | 7200 (26.1%) |
| Mineralocorticoid receptor antagonists^‡^ (%) | 1026 (5.9%) | 5512 (16.2%) | 3578 (12.9%) |
| Signs and symptoms^‡^ (%) | 4614 (26.7%) | 14 985 (44.0%) | 11 224 (40.7%) |
| (NT-pro)BNP measured^‡^ (%) | 103 (0.6%) | 322 (0.9%) | 797 (2.9%) |
| (NT-pro)BNP elevated^‡^ (%) | 81 (0.5%) | 240 (0.7%) | 567 (2.1%) |
| Cardiac imaging performed^‡^ (%) | 610 (3.5%) | 3102 (9.1%) | 6738 (24.4%) |
| Cardiac care^‡^ (%) | 491 (2.8%) | 2966 (8.7%) | 6327 (23.0%) |
| Referral to HF specialist^‡^ (%) | 53 (0.3%) | 351 (1.0%) | 662 (2.4%) |
| CPRD=Clinical Practice Research Datalink; HES=Hospital Episode Statistics; ONS=Office for National Statistics  *assessed by index of multiple deprivation † measurement closest to and within 6 months before or on the date of heart failure diagnosis. § denotes prior medical history of given comorbidity. ‡ denotes present or prescribed ± 6 months of index date for heart failure diagnosis. \|\| cardioselective betablockers include metoprolol, carvedilol, or bisoprolol. | | | |

| **Supplementary Table 8.** **Association between patient characteristics and 5-year all-cause mortality after index heart failure diagnosis, adjusted for age, sex and stratified by primary care practice.** | | | |
| --- | --- | --- | --- |
|  | **Source of heart failure record** | | |
|  | **CPRD** | **CPRD and HES** | **HES** |
|  | **Primary care diagnosis of HF only** | **Primary care diagnosis of HF and at least one HF hospitalisation ever** | **Hospitalised for HF without primary care diagnosis of HF ever** |
| **Variables** | **Hazard Ratio (95% CI)** | **Hazard Ratio (95% CI)** | **Hazard Ratio (95% CI)** |
| Age, per 10 year increase | 1.68 (1.65 – 1.72) | 1.54 (1.51 – 1.57) | 1.48 (1.46 – 1.50) |
| Sex, women vs men | 0.78 (0.75 – 0.81) | 0.89 (0.86 – 0.92) | 0.89 (0.87 – 0.92) |
| Current smoker vs never | 1.40 (1.31 – 1.49) | 1.27 (1.20 – 1.35) | 1.31 (1.25 – 1.38) |
| BMI (per 1 kg/m^2^ increase) | 0.97 (0.96 – 0.97) | 0.98 (0.97 – 0.99) | 0.98 (0.98 – 0.99) |
| Systolic blood pressure (per 10mmHg increase) | 0.95 (0.94 – 0.96) | 0.95 (0.95 – 0.96) | 0.96 (0.96 – 0.98) |
| Atrial fibrillation | 0.96 (0.92 – 1.00) | 0.96 (0.93 – 1.00) | 0.97 (0.94 – 1.00) |
| Diabetes | 1.34 (1.26 – 1.43) | 1.39 (1.33 – 1.46) | 1.25 (1.20 – 1.29) |
| COPD | 1.43 (1.36 – 1.50) | 1.26 (1.21 – 1.32) | 1.20 (1.16 – 1.24) |
| Depression | 1.10 (1.05 – 1.15) | 1.07 (1.03 – 1.12) | 1.02 (0.98 – 1.07) |
| Cancer | 1.43 (1.37 – 1.49) | 1.33 (1.28 – 1.39) | 1.31 (1.26 – 1.35) |
| Creatinine (per 30 units increase) | 1.09 (1.08 – 1.11) | 1.07 (1.06 – 1.08) | 1.05 (1.04 – 1.06) |
| Haemoglobin (per 1 mmol/L increase) | 0.89 (0.88 – 0.90) | 0.90 (0.89 – 0.91) | 0.95 (0.95 – 0.96) |
| CPRD=Clinical Practice Research Datalink; HES=Hospital Episode Statistics; ONS=Office for National Statistics  HR denotes hazard ratio, follow up period was 1997-2010 | | | |


**FIGURES**

**Supplementary Figure 1. Flow chart of in- and exclusion CALIBER heart failure patients**

**Supplementary Figure 2. Diagram of patient groups based on electronic health record source**

**Supplementary Figure 3. Histogram of days to primary care record following index event of acute HF hospital admission recorded in HES.**

**
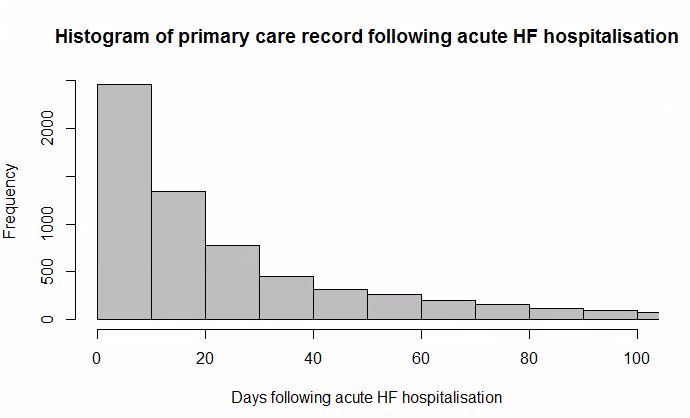
**

**Supplementary Figure 4. Number of patients with hospital admissions and re-admissions in Hospital Episode Statistics (HES) from 1^st^ Jan 1997 to 26 March 2010.**
